# Comment on Yamazaki et al. Long-Term Effects of Tolvaptan in Autosomal Dominant Polycystic Kidney Disease: Predictors of Treatment Response and Safety over 6 Years of Continuous Therapy. *Int. J. Mol. Sci.* 2024, *25*, 2088

**DOI:** 10.3390/ijms27167410

**Published:** 2026-08-19

**Authors:** Liliana Italia De Rosa, Martina Catania, Kristiana Kola, Giuseppe Vezzoli, Matteo Brambilla Pisoni, Sara Farinone, Paolo Manunta, Maria Teresa Sciarrone Alibrandi

**Affiliations:** 1 School of Nephrology, Vita Salute San Raffaele University, 20132 Milan, Italy; derosa.liliana@hsr.it (L.I.D.R.); catania.martina@hsr.it (M.C.); kola.kristiana@hsr.it (K.K.); vezzoli.giuseppe@hsr.it (G.V.); manunta.paolo@hsr.it (P.M.); 2 O.U. Nephrology and Dialysis, San Raffaele Scientific Institute, 20132 Milan, Italy; brambilla.matteo@hsr.it; 3 Clinical and Health Psychology Unit, San Raffaele Scientific Institute, 20132 Milan, Italy; farinone.sara@hsr.it

## 1. Introduction

In response to the article [1], we wish to highlight our experience as a leading referral center in Italy for the treatment of autosomal dominant polycystic kidney disease (ADPKD) with a multidisciplinary approach [2]. Over the past 15 years, we have managed approximately 2000 patients from across the country, establishing ourselves as a key institution in advancing care for this complex disease.

ADPKD is a chronic, progressive disorder that significantly impairs quality of life. Patients frequently endure symptoms such as chronic pain, hypertension, recurrent infections, and eventual kidney failure [3]. Addressing these complications necessitates lifelong interventions, which can be physically and emotionally burdensome [4]. Tolvaptan, a cornerstone therapy for slowing disease progression, introduces additional challenges due to its side effect profile. Optimizing the management of Tolvaptan therapy is crucial, not only to minimize complications but also to reduce the overall treatment burden on patients already facing considerable challenges in their daily lives.

We have actively participated in landmark studies, including the TEMPO 3:4 trial and the REPRISE study [5,6], as well as their extension studies [7]. Currently, we manage 80 patients on chronic Tolvaptan therapy. Our clinical findings align with those in the literature, particularly regarding the elevation of hepatic enzymes, a well-documented side effect of Tolvaptan.

However, we wish to draw attention to a critical and often-overlooked issue: severe hyponatremia associated with long-term Tolvaptan therapy. Although uncommon, this complication has significant clinical implications and can impact patient outcomes if not promptly identified and managed.

## 2. A Paradoxical Complication of Therapy

Tolvaptan, a vasopressin V2-receptor antagonist, promotes aquaresis by increasing urine output and reducing urine osmolality [8]. This mechanism is beneficial for managing conditions such as refractory hyponatremia, for which Tolvaptan was originally developed. In ADPKD, Tolvaptan inhibits vasopressin-induced cyclic AMP production in renal tubular cells, thereby reducing cyst growth and slowing kidney function decline [1,5]. Paradoxically, however, prolonged high-dose therapy may lead to severe hyponatremia, a complication that is underreported in shorter studies and clinical trials due to its tendency to manifest only with extended use [9].

In our cohort, severe hyponatremia (serum sodium levels <125 mmol/L) was observed in 5% of patients (4 out of 80) receiving chronic Tolvaptan therapy. All affected patients were on the maximum dose of 120 mg, with therapy durations of at least three years. In one case, a patient who had been on therapy for over five years experienced a serum sodium level as low as 118 mmol/L, presenting with gastrointestinal and neurological symptoms. Effective management included dietary sodium supplementation, reduction in fluid intake, and, in one case, temporary discontinuation of Tolvaptan.

## 3. To Mitigate the Risk of Severe Hyponatremia, We Propose the Following Strategies

A multidisciplinary approach is essential for effectively managing severe hyponatremia and improving the overall safety and tolerability of chronic Tolvaptan therapy. The following strategies emphasize the importance of collaboration among healthcare professionals:

Enhanced patient education: Clear and ongoing guidance on balancing fluid intake and dietary sodium is crucial. Patients should be informed about the risks of excessive water consumption, particularly those prone to anxiety about therapy compliance. In this context, the role of the psychologist is pivotal [2,10]. Psychologists can assist patients in managing anxiety related to chronic disease, address dysfunctional behaviors associated with fluid management, and support adherence to treatment protocols, ultimately reducing the risk of complications.

Frequent monitoring: Increased frequency of laboratory assessments is necessary, particularly for patients on maximum Tolvaptan doses and in the later stages of therapy. Monthly evaluations of serum sodium levels, urine specific gravity, and hydration status are recommended for high-risk individuals.

Integrated follow-up plans: Comprehensive hydration protocols and dietary assessments should be part of routine care. The involvement of a dietitian is critical for designing personalized dietary plans that ensure adequate sodium intake without compromising blood pressure control. Multidisciplinary collaboration among nephrologists, dietitians, and psychologists [2] ensures a holistic approach to therapy, addressing both clinical and psychosocial aspects of care.

## 4. Conclusions

Severe hyponatremia is a rare but clinically significant complication of prolonged high-dose Tolvaptan therapy. Effective management requires a proactive approach involving patient education, dietary modifications, and close monitoring. Integrating professionals such as dietitians and psychologists into the multidisciplinary team not only improves the safety and tolerability of therapy but also provides essential support to address the emotional and behavioral challenges faced by ADPKD patients.

## Data Availability

No new data were created or analyzed in this study. Data sharing is not applicable to this article.
